# Toxic Dimethylarginines: Asymmetric Dimethylarginine (ADMA) and Symmetric Dimethylarginine (SDMA)

**DOI:** 10.3390/toxins9030092

**Published:** 2017-03-06

**Authors:** You-Lin Tain, Chien-Ning Hsu

**Affiliations:** 1 Department of Pediatrics, Kaohsiung Chang Gung Memorial Hospital and Chang Gung University College of Medicine, Kaohsiung 833, Taiwan; tainyl@hotmail.com; 2 Institute for Translational Research in Biomedicine, Kaohsiung Chang Gung Memorial Hospital and Chang Gung University College of Medicine, Kaohsiung 833, Taiwan; 3 Department of Pharmacy, Kaohsiung Chang Gung Memorial Hospital, Kaohsiung 833, Taiwan; 4 School of Pharmacy, Kaohsiung Medical University, Kaohsiung 807, Taiwan

**Keywords:** alanine-glyoxylate aminotransferase-2, asymmetric dimethylarginine, cardiovascular disease, chronic kidney disease, dimethylarginine dimethylaminohydrolase, nitric oxide, non-proteinogenic amino acid, protein arginine methyltransferase, symmetric dimethylarginine, uremic toxins

## Abstract

Asymmetric and symmetric dimethylarginine (ADMA and SDMA, respectively) are toxic, non-proteinogenic amino acids formed by post-translational modification and are uremic toxins that inhibit nitric oxide (NO) production and play multifunctional roles in many human diseases. Both ADMA and SDMA have emerged as strong predictors of cardiovascular events and death in a range of illnesses. Major progress has been made in research on ADMA-lowering therapies in animal studies; however, further studies are required to fill the translational gap between animal models and clinical trials in order to treat human diseases related to elevated ADMA/SDMA levels. Here, we review the reported impacts of ADMA and SDMA on human health and disease, focusing on the synthesis and metabolism of ADMA and SDMA; the pathophysiological roles of these dimethylarginines; clinical conditions and animal models associated with elevated ADMA and SDMA levels; and potential therapies against ADMA and SDMA. There is currently no specific pharmacological therapy for lowering the levels and counteracting the deleterious effects of ADMA and SDMA. A better understanding of the mechanisms underlying the impact of ADMA and SDMA on a wide range of human diseases is essential to the development of specific therapies against diseases related to ADMA and SDMA.

## 1. Introduction

The dimethylarginines, asymmetric dimethylarginine (ADMA) and symmetric dimethylarginine (SDMA), were first isolated from human urine in 1970 [1]. Among the guanidine compounds listed as uremic toxins [2], ADMA and SDMA and have been increasingly recognized as putative toxic non-proteinogenic amino acids in a wide range of human diseases over the past decades [3,4,5,6,7,8,9,10,11].

The biological relevance of ADMA as an endogenous inhibitor of nitric oxide synthase (NOS) was first described by Vallance et al. [3]. Although less attention has been paid to SDMA, Bode-Boger et al. were the first to report in vitro inhibitory effects of nitric oxide (NO) production by SDMA [12]. Given that NO has pleiotropic bioactivities, it is not surprising that a variety of important biological functions are regulated by ADMA and SDMA. Emerging clinical and experimental evidence indicates that ADMA and SDMA are involved in the pathophysiology of endothelial dysfunction [13], atherosclerosis [4], oxidative stress [14,15], inflammation [16,17], uremia [8], apoptosis, [18], autophagy [19], and impaired immunological function [20].

This review provides an overview of potential pathophysiological roles for both ADMA and SDMA in human health and disease, with emphasis on the synthesis and metabolism of ADMA and SDMA, the pathophysiology of dimethylarginines, clinical conditions with elevated ADMA and SDMA concentrations, and potential therapies to reduce ADMA and SDMA levels. 

## 2. Synthesis and Metabolism of ADMA and SDMA

### 2.1. Synthesis of ADMA and SDMA

Non-proteinogenic amino acids are those not naturally encoded or found in the genetic code of organisms. Some of them are formed by post-translational modification of the side chains of proteinogenic amino acids present in proteins. Protein-incorporated ADMA is formed by post-translational methylation: two methyl groups are placed on one of the terminal nitrogen atoms of the quanidino group of arginine in proteins by a family of protein arginine methyltransferases (PRMTs) [21]. SDMA, with one methyl group positioned on each of the terminal guanidine nitrogens, is a structural isomer of ADMA. To date, nine human PRMT genes have been cloned and PRMTs are divided into enzymes with type I, type II, or type III activity. Type I PRMTs (PRMT-1, -3, -4, -6, and -8) generate ADMA, whereas type II PRMTs (PRMT-5 and -9) produce SDMA. Although peptidyl arginine deiminases (PADs) can block methylation of arginine residues within proteins by converting them to citrulline [22], PADs are not demethylases. The first arginine demethylase, JMJD6, has been identified [23]; however, a direct role for JMJD6 in the demethylation of protein-incorporated ADMA and SDMA has not been validated [24].

### 2.2. Metabolism of ADMA and SDMA

Free ADMA and SDMA are released following proteolysis. A healthy adult produces 60 mg (~300 μmol) ADMA per day, of which approximately 20% is excreted in urine via the kidneys [25]. In contrast to ADMA, SDMA is present at only ~50% of the levels of ADMA and the elimination of SDMA is largely dependent on urinary excretion. Free ADMA and SDMA share a common transport process with l-arginine and as such can be moved into or out of cells via the cationic amino acid transporter (CAT) family [26]. Circulating ADMA can hence be transported to major organs such as the kidney, brain, and liver for enzymatic degradation. To date, three enzymes have been reported to metabolize ADMA: dimethylarginine dimethylaminohydrolase-1 (DDAH-1) and -2 (DDAH-2) as well as alanine-glyoxylate aminotransferase 2 (AGXT2), among which DDAHs metabolize ADMA to citrulline and dimethylamine. Similarly, ADMA can also be transaminated by the enzyme AGXT2 to α-keto-δ-(*N*^G^,*N*^G^-dimethylguanidino) valeric acid (DMGV) [27]. Accordingly, plasma and tissue ADMA levels are highly dependent on factors that affect the expression and activity of DDAHs and AGXT2. Several mechanisms of inhibition of the expression and/or activity of DDAHs have been described [28], including hyperglycemia [29], oxidative stress [30], and angiotensin II administration [31]. Unlike DDAHs, AGXT2, a mitochondrial aminotransferase expressed primarily in the kidney, can metabolize not only ADMA but also SDMA [27]. This AGXT2-mediated pathway of dimethylarginine metabolism has, however, received relatively little attention and the metabolic pathway of this mechanism is still poorly understood. Only one report has shown that D-β-aminoisobutyric acid can inhibit Agxt2-mediated metabolism of ADMA and SDMA [32].

**Figure 1:**
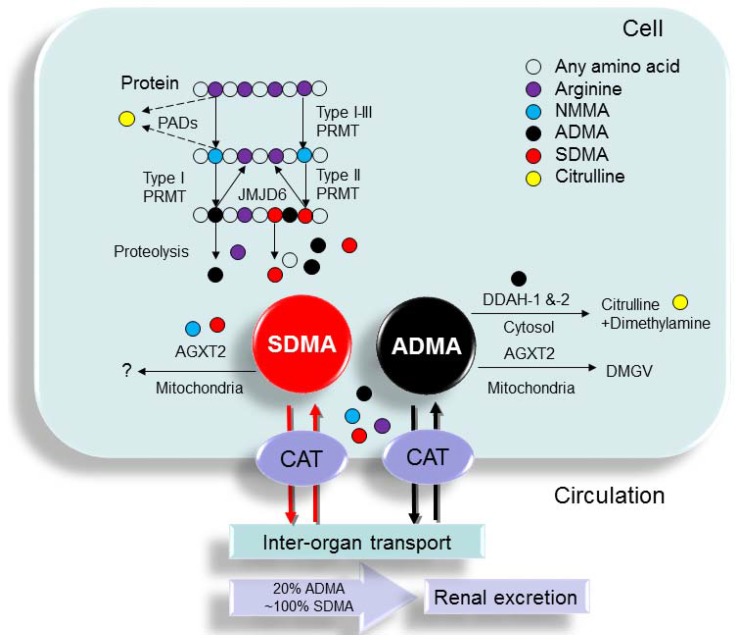
Schema outlining the synthesis and metabolism of ADMA and SDMA. Protein arginine (purple circle) methylation is performed by a family of enzymes termed protein arginine methyltransferases (PRMTs), which methylate protein-incorporated l-arginine residues to generate protein-incorporated N^G^ monomethyl-l-arginine (NMMA; blue circle). Type I PRMTs generate asymmetric dimethylarginine (ADMA; black circle) and type II PRMTs convert NMMA to symmetric dimethylarginine (SDMA; red circle). Protein-incorporated l-arginine residues can also be converted to citrulline (yellow circle) by peptidylarginine deaminases (PADs), thereby blocking methylation on the arginine residue. Upon proteolytic cleavage of arginine-methylated proteins, free ADMA and SDMA are released into the cytoplasm. ADMA and SDMA can be moved out of the cells via cationic amino acid transporter (CAT) and transported to other organs or excreted in urine. ADMA can be converted to l-citrulline and dimethylamine by dimethylarginine dimethylaminohydrolase-1 (DDAH-1) and -2 (DDAH-2). Alanine-glyoxylate aminotransferase 2 (AGXT2), a mitochondrial aminotransferase expressed primarily in the kidney, can metabolize ADMA as well as SDMA. ADMA can be transaminated by the enzyme AGXT2 to α-keto-δ-(*N*^G^,*N*^G^-dimethylguanidino) valeric acid (DMGV).

In addition to ADMA and SDMA, a third methylarginine residue—N^G^ monomethyl-l-arginine (NMMA)—is produced in mammals. Since the levels of NMMA are much lower than those of ADMA and SDMA, very little information is available regarding its pathophysiological role in clinical conditions, except that it can function as a NOS inhibitor [28]. The biochemical pathways related to the synthesis and metabolism of SDMA and ADMA are illustrated in Figure 1.

### 2.3. Quantification of ADMA and SDMA

Since ADMA and SDMA show a very narrow range of normal concentrations, high analytical precision is mandatory to distinguish between normal and slightly elevated concentrations [33]. So far, analytical techniques for the quantification of ADMA and SDMA levels include high-performance liquid chromatography (HPLC) [34], gas chromatography (GC)–mass spectrometry (MS) [35], liquid chromatography with mass spectrometric detection (LC-MS and LC-MS/MS) [36,37], ultrahigh performance liquid chromatography (UPLC)-MS/MS [38], and enzyme-linked immunosorbent assay (ELISA) [39]. Since ADMA and SDMA are structural isomers of each other with an identical molecular weight of 202.1, chromatographic separation using HPLC with ultra violet (UV), radioimmunoassay, and fluorescence (FL) detection was shown to be required. HPLC-based methods are the most widely used techniques for assessing ADMA and SDMA levels in biological fluids such as plasma, urine, and tissue homogenate. These HPLC methods, however, are very time consuming. Although MS-based methods are more sensitive, ADMA and SDMA exhibit different patterns of dissociation between various MS systems. ELISA methods, furthermore, tend to overestimate ADMA concentrations [40,41]; there is only moderate correlation between quantification by ELISA compared with that by UPLC-MS/MS for both ADMA and SDMA [38]. Standardized analytical techniques are required in order for ADMA and SDMA levels to be reliably assessed on a routine basis in clinical practice.

## 3. Clinical Conditions Associated with Elevated ADMA and SDMA Levels

### 3.1. ADMA and SDMA: From Uremic Toxins to CVD Risk Factors

Numerous clinical studies have demonstrated elevated ADMA and SDMA levels in a wide spectrum of human diseases [3,4,5,6,7,8,9,10,11]. Since ADMA and SDMA are both uremic toxins [2], the pathophysiological relevance of these two toxic non-proteinogenic amino acids has been extensively investigated in chronic kidney disease (CKD) and end-stage renal disease (ESRD) [42]. Although nearly all studies show that circulating ADMA levels are elevated in patients with CKD, even before a reduction in glomerular filtration rate (GFR), ADMA is not considered a prognosis biomarker in patients with renal disease [42]. A meta-analysis including 2136 patients from 18 studies, however, demonstrated a strong correlation between SDMA and renal function [43]. Zoccali et al. were the first to report the association between circulating ADMA and cardiovascular disease (CVD) and mortality in patients with renal disease [44]. Since then, a number of studies have linked circulating ADMA to CVD risk and mortality in many different study populations. A recent meta-analysis based on 30 studies with 30,624 subjects and 3396 incident CVD events reported that the relative risks for all-cause mortality associated with CVD were 1.52 (1.37–1.68) and 1.33 (1.22–1.45) for high and low ADMA concentrations, respectively [45]. Additionally, high vs. low levels of SDMA were shown to be associated with 31% and 36% increased risk for all-cause mortality and CVD events, respectively [45]. 

### 3.2. Clinical Conditions Associated with Elevated ADMA Levels

To date, the list of clinical conditions in which elevated ADMA levels are found continues to grow. Here, we summarize studies previously reviewed [25,42,43,44,45,46] and highlight new data documenting associations between elevated ADMA levels and clinical conditions in specific patient populations. As shown in Table 1, elevated circulating ADMA concentrations have been described in a variety of diseases across different age and sex groups [3,42,46,47,48,49,50,51,52,53,54,55,56,57,58,59,60,61,62,63,64,65,66,67,68,69,70,71,72,73,74,75,76,77,78,79,80,81,82,83,84,85,86,87,88,89,90]. Differences in ADMA concentrations between sexes are small [33], whereas difference between different age groups do exist. In adults, plasma ADMA levels increase with age and the mean plasma concentration of ADMA for a healthy adult is between 0.4 and 0.6 μM [40]. ADMA levels vary by almost two-fold across the geriatric population [91] and in neonates, venous cord blood ADMA levels are markedly elevated (~1.06 μM) and fall significantly close to the normal adult value by the second postnatal day (~0.66 μM) [92]. ADMA levels are higher in children than in adults and levels diminish from birth until around 25 years of age with a mean decrease rate of 15 nM per year [93]. Although ADMA levels are the highest in geriatric and neonatal populations, whether this U-shaped relationship between normal ADMA levels and age relates to renal function remains unclear.

In addition to renal disease [46], increased plasma ADMA levels are associated with clinical conditions mainly associated with endothelial dysfunction such as hypertension [48], peripheral arterial occlusive disease [49], hypercholesterolemia [50], preeclampsia [52], diabetes mellitus [53,59], stroke [55], obesity [60], coronary artery disease [64], polycystic ovary syndrome [67], and sickle cell disease [71]. Many diseases affect both women and men alike; however, some diseases occurring at a higher frequency in women (e.g., systemic lupus erythematous) or affecting only women (e.g., preeclampsia [52], polycystic ovary syndrome [67], and primary dysmenorrhea [73]) have been linked with elevated ADMA concentrations. Additionally, some pediatric diseases such as prematurity [64], congenital urea cycle enzyme defects [69], and transient tachypnea in newborns [88] are associated with elevated plasma ADMA levels. As shown in Table 1, patients with ASL deficiency have been shown to have elevated ADMA levels [69]. It has been noted that hypertension is over-represented in persons with argininosuccinate lyase (ASL) deficiency, a urea cycle disorder [94]. Since ADMA levels are highly correlated with CVD outcome and the occurrence of preclinical CVD during childhood is rare, consideration should be given to elucidating the pathophysiological role of ADMA and to determining the long-term CV outcome in these pediatric diseases. Moreover, it is important to note that diseases reported with elevated ADMA concentrations exhibit remarkable variability across different subspecialties. The extent to which the ADMA affects human health warrants further investigation. 

### 3.3. Clinical Conditions Associated with Elevated SDMA Levels

Despite less attention having been paid to SDMA than to ADMA, there is still a substantial body of research linking elevated SDMA levels to clinical conditions (Table 2) [44,48,56,57,63,64,95,96,97,98,99,100,101,102,103]. As already mentioned, a meta-analysis study showed that SDMA levels correlate well with renal function [44]. SDMA has furthermore been considered a marker of acute kidney injury [104]. As in the case of ADMA, elevated SDMA levels have been reported in clinical conditions related to endothelial dysfunction such as hypertension [56], coronary artery disease [64], diabetes mellitus [96], preeclampsia [99], stroke [100], polycystic ovary syndrome [101], and hyperuricemia [102]. Similar to ADMA, SDMA has been shown to be able to predict all-cause mortality and CVD events, which is independent of renal function [44].

### 3.4. Causal Link between the Plasma Levels of ADMA or SDMA and Clinical Outcome

The list of clinical conditions associated with elevated ADMA and SDMA levels continues to grow; however, these clinical observations only describe relationships and do not allow for interpretation of the causality. A few human studies have demonstrated that the administration of ADMA to healthy volunteers leads to endothelial dysfunction, increased vascular resistance and arterial blood pressure, as well as decreased cardiac output [105,106]. Although dimethylarginine levels have been analyzed in different tissue fluid samples in specific populations [107,108], almost all studies demonstrating an association between ADMA or SDMA and clinical diseases referred to blood plasma levels of ADMA or SDMA and not tissue levels. Although many studies have demonstrated that plasma ADMA or SDMA levels are elevated in patients with a broad range of disorders, intracellular ADMA and SDMA levels in these disorders have not been well studied. Human tissue samples are difficult to attain and thus in vitro studies may be advantageous for studying intracellular dimethylarginine regulation. It furthermore remains to be determined whether reduced levels of ADMA and SDMA result in reduced CVD risk and improved outcome in the above mentioned diseases. It stands to reason that much of our knowledge on potential therapies involving lowering ADMA and SDMA in specific diseases is based on animal research.

## 4. Pathophysiology of ADMA and SDMA

### 4.1. ADMA and SDMA: Inhibition of NO Synthesis

The most well-known effect of ADMA and SDMA is the inhibition of NO production. At physiological extracellular l-arginine and ADMA concentrations, intracellular NOS is well saturated with the substrate l-arginine and physiological levels of NO are produced. In the presence of pathological concentrations of ADMA, NOS activity decreases, resulting in a reduction of NO. Cellular ADMA levels can be 5- to 20-fold higher than those in the plasma and can fall in the range known to inhibit NOS [26]. Under such conditions, the addition of exogenous l-arginine shifts intracellular ADMA and restores the physiological l-arginine:ADMA ratio to a level that preserves sufficient NO production. The state of NOS activation or inhibition therefore depends on the local intracellular l-arginine:ADMA ratio. SDMA, on the other hand, does not directly inhibit NOS but is a competitive inhibitor of l-arginine transport [12].

### 4.2. Tissue ADMA and SDMA Concentrations

Although many human diseases, including CVD, are associated with increased plasma levels of ADMA and SDMA, little is known to date about intracellular levels. Elevated ADMA levels in the kidneys develop early on, even before the onset of hypertension in four-week-old spontaneously hypertensive rats (SHRs) [109]. Moreover, elevated levels of ADMA in the lung were observed in the hypertensive stage in SHRs [110]. A previous report furthermore demonstrated that ADMA concentrations are increased in the aortas of obese diabetic mice [111] and in a streptozotocin (STZ)-induced diabetic mother rat model, offspring developed hypertension and kidney disease, which is associated with elevated renal levels of ADMA [112]. These findings suggest a role for intracellular ADMA in the development of CVD.

A recent report showed strong differences in ADMA and SDMA levels between different tissues from mice [113]: the concentrations of ADMA and SDMA are high in the kidney, liver, pancreas, and spleen; intermediate in the lung and heart; and lowest in the brain. The differences in ADMA abundance across different tissues may be due to differential expression of DDAHs in various tissues. Data from a DDAH-1 and -2 knockdown model showed that ADMA is regulated by DDAH-1, which is expressed at sites of ADMA metabolism in the kidney cortex and liver, whereas NO is regulated primarily by DDAH-2, which is expressed strongly in the blood vessels [114]. Although DDAH-1 is also highly expressed in the kidney and liver [115], both these organs have been reported as major sites for the metabolism of excessive circulating ADMA [116]. Accordingly, ADMA concentrations are high in the liver and kidney. Since DDAH-1 is abundantly expressed in the brain at sites of neuronal NOS expression [98], ADMA may be expeditiously metabolized by DDAH-1 in the brain. Intracellular ADMA can, moreover, be regulated differentially in different tissues in the same disease model. Plasma, hepatic, and renal ADMA levels have been evaluated simultaneously in young rats two weeks after bile-duct ligation (BDL), a commonly used cholestatic liver disease model [117]. The increase in circulating ADMA results primarily from increased synthesis of ADMA (by increased PRMT1 abundance) in the liver. The metabolism of ADMA is unaltered in the damaged liver, indicating unaltered DDAH expression and/or activity in the liver. The decreased renal DDAH activity, however, suggests that the kidney is unable to metabolize excessive ADMA. Unlike liver and kidney ADMA levels, ADMA levels in brain cortex of young BDL rats were not altered [118]. These findings highlight the importance of studying tissue ADMA levels instead of plasma ADMA levels: changes in plasma ADMA do not correlate with intracellular ADMA levels in different tissues. It is therefore important to note that systemic and tissue ADMA levels must be assessed simultaneously to elucidate the relative importance of different mechanisms regulating ADMA homeostasis.

### 4.3. ADMA: Multifunctional Effects

ADMA can uncouple NOS isoenzymes to produce superoxide, contributing to the burden of oxidative stress [119]. Furthermore, three transcriptomic studies have suggested that ADMA may contribute to a wide range of pathologies [120,121,122]. Using microarray technology, Smith et al. first reported that >50 genes were altered in endothelial cells in response to pathological concentrations of ADMA [120] and BMP signaling and enzymes involved in the arginine methylation pathway were also shown to be significantly regulated by ADMA levels. Next-generation sequencing (NGS) was subsequently used to assess the renal transcriptome response to ADMA in the developing kidney. A total of 1221 differentially expressed genes (DEGs) (735 up- and 486 down-regulated genes in ADMA-treated vs. control samples) were identified. Thirteen significantly related Kyoto Encyclopedia of Genes and Genomes (KEGG) pathways were identified in the developing kidney treated with ADMA, including ribosome, cytokine-cytokine receptor interaction, chemokine signaling pathway, neuroactive ligand-receptor interaction, arachidonic acid metabolism, intestinal immune network for IgA production, systemic lupus erythematosus, toll-like receptor signaling pathway, NOD-like receptor signaling pathway, tyrosine metabolism, and the MAPK signaling pathway [112,122]. A recent report furthermore showed that serum starvation profoundly altered the gene expression of LoVo tumor cells by microarray analysis and that ADMA could restore most of the changes at the transcriptional level [122]. These findings imply that pathophysiological concentrations of ADMA can elicit significant changes at the gene expression level and that these changes may be exerted in a NO pathway-independent manner.

### 4.4. SDMA: Pro-Inflammatory and Pro-Oxidant Properties

Compared with ADMA, little attention has been paid to the pathophysiological role of SDMA. In addition to inhibiting NO production [12], SDMA may have pro-inflammatory effects [123]. SDMA has been reported to induce the expression of CD11a, CD11b, and CD14 in monocytes as well as CD18 expression in granulocytes to enhance the differentiation and adhesion capacity of leukocytes to the endothelium. Additionally, SDMA may induce reactive oxygen species (ROS) via store-operated calcium influx in monocytes [15] and enhancement of NADPH-oxidase via the activation of endothelial Toll-like receptor-2 [124]. Accordingly, SDMA may be involved directly or indirectly in the pathogenesis of CVD because of its pro-inflammatory and pro-oxidant properties.

## 5. Potential Therapies for Reducing ADMA and SDMA Levels

To date, there is a lack of potential therapeutic strategies against elevated ADMA and SDMA levels in various diseases. Since both dimethylarginines are water-soluble uremic toxins [2], it would be logical to consider dialysis as a potential means of decreasing of circulating ADMA and SDMA levels. A previous study showed that a single dialysis session reduced ADMA and SDMA plasma levels by 23% and 40%, respectively [125], and the removal of ADMA and SDMA by dialysis seems to be hampered by complex kinetics of these two uremic toxins. Dialysis is furthermore not suitable for non-uremia patients in clinical practice.

Since approximately 80% of ADMA is metabolized in the body, alternative therapeutic approaches have been assessed; however, to date, a specific ADMA-lowering agent is still not available. As previously reviewed by us and others [6,122,126], a few drugs have been reported to lower ADMA levels in clinical studies. These include angiotensin-converting enzyme inhibitors, angiotensin receptor blockers, fenofibrate, oral contraceptives, folic acid, metformin, and α-lipoic acid. Despite a partial reduction of plasma ADMA levels by these therapies, the underlying mechanisms of their ADMA-lowering effects are still unclear. Since PRMTs control ADMA production and as DDAHs and AGXT2 regulate its metabolism, the discovery and application of specific PRMT inhibitors, DDAH activators, or AGXT2 activators may represent potential therapeutic strategies. The development of specific PRMT inhibitors, DDAH activators, and AGXT2 activators for ADMA suppression, however, remains a challenging area of research [7,127,128]. Currently, numerous therapies have been shown to reduce ADMA concentrations in a wide range of animal models (Table 3) [110,112,129,130,131,132,133,134,135,136,137,138,139,140,141,142,143,144,145,146,147,148,149,150,151,152,153,154,155,156,157,158,159,160,161,162,163,164,165,166,167,168]. Some of the major approaches include the restoration of the imbalance between l-arginine and ADMA, the regulation of DDAH enzymes and/or activity, and the inhibition of PRMT expression.

Since l-arginine is the substrate for NOS-mediated production of NO, l-arginine and l-citrulline (the precursor of l-arginine) supplementation have been reported to reduce ADMA and increase NO bioavailability in a variety of models with elevated ADMA levels [112,134,149,150]. A number of animal studies have furthermore indicated that pioglitazone [131], farnesoid X receptor agonist [132], telmisartan [136], resveratrol [137], melatonin [117,138], atorvastatin [143], shichimotsukokato [144], vitamin E [145], salvianolic acid A [146], *N*-acetylcysteine [148], oxymatrine [152], metformin [154], epigallocatechin-3-gallate [166], and rosuvastatin [168] can increase the activity and/or expression of DDAHs and thereby reduce ADMA levels. On the other hand, telmisartan [136], glucagon-like peptide-1 receptor agonist [147], epigallocatechin-3-gallate [166], and rosuvastatin [168] may reduce ADMA levels via decreased PRMT-1 expression. However, whether these ADMA-lowering therapies not only reduce circulating ADMA levels but also ADMA levels in target organs requires further clarification. Further investigation is also required to determine the mechanisms of other effective ADMA-lowering agents and to clarify whether these agents can be applied to different disease models associated with elevated ADMA levels.

## 6. Conclusions

Since the first isolation of ADMA and SDMA from human urine in 1970, there has been substantial evidence revealing the significance of these two non-proteinogenic amino acids in human health and diseases. ADMA and SDMA are known uremic toxins and the most well-known effect of these two toxic amino acids is the suppression of NO production. High plasma ADMA or SDMA concentrations not only predict all-cause mortality and CVD events, but are also relevant to a broad range of diseases. Although significant progress has been made in research on ADMA and SDMA, there is a need for a simple and sensitive method for measuring ADMA and SDMA levels simultaneously on a routine basis. The development of specific pharmacological therapy for lowering ADMA and SDMA levels in target organs is still a far-off goal and requires a deeper understanding of the multifunctional effects of ADMA and SDMA in target organs that induce a variety of diseases. Accordingly, there is an urgent need for the elucidation of unknown biological functions and for the development of effective strategies for treating diseases associated with high levels of ADMA and SDMA.

## Figures and Tables

**Table 1:** Clinical conditions associated with elevated ADMA levels.

Patient Population	*N*	Correlation with Clinical Outcome	Year of First Report	Ref.
CKD/ESRD	>1500	ND	1992	[3,46]
Schizophrenia	16	ND	1996	[47]
Childhood hypertension	38	ND	1997	[48]
Peripheral arterial occlusive disease (PAOD)	77	ND	1997	[49]
Hypercholesteremia	49	ND	1998	[50]
Congestive heart failure	84	ADMA positively correlates with severity of heart failure	1998	[51]
Preeclampsia	12	ND	1998	[52]
Type 2 diabetes	50	ADMA correlates with brachial arterial dilation	2000	[53]
Congenital heart disease (CHD)	20	Elevated ADMA in CHD with pulmonary hypertension	2001	[54]
Stroke	52	ADMA correlates with homocysteine level	2001	[55]
Hyperthyroidism	19	ADMA correlates with free T4 level	2002	[56]
Critical illness in intensive care unit	52	ADMA increases risk for ICU death	2003	[57]
Liver cirrhosis	11	ND	2004	[58]
Type 1 diabetes	408	ADMA correlates with CVD events	2004	[59]
Obesity	563	ND	2004	[60]
Systemic lupus erythematous	107	ADMA correlates with CVD events	2005	[61]
Idiopathic pulmonary arterial hypertension	57	ND	2005	[62]
Hepatorenal syndrome	11	ND	2006	[63]
Coronary artery disease	145	ADMA correlates with homocysteine level; ADMA negatively correlates with GFR	2006	[64]
Prematurity	19	Elevated ADMA in male premature	2006	[65]
Systemic sclerosis	21	Elevated ADMA in diffuse systemic sclerosis	2006	[66]
Polycystic ovary syndrome (PCOS)	106	ND	2008	[67]
Obstructive sleep apnea-hypopnea syndrome (OSAHS)	34	ND	2008	[68]
Congenital urea cycle enzyme defects	15	Elevated ADMA in argininosuccinate synthase (ASS) deficiency and argininosuccinate lyase (ASL) deficiency	2009	[69]
Rheumatiod arthritis (RA)	25	ND	2009	[70]
Sickle cell disease (SCD)	177	ADMA correlates with mortality	2009	[71]
Congenital portosystemic venous shunt (PSVS)	14	ND	2010	[72]
Primary dysmenorrhea	33	ND	2010	[73]
Inflammatory bowel diseases (IBD)	63	ADMA correlates with Crohn’s disease activity	2010	[74]
Asthma	17	ND	2011	[75]
Nonalcoholic fatty liver disease (NAFLD)	35	ND	2011	[76]
Psoriatic arthritis	22	ADMA correlates with coronary flow reserve	2011	[77]
Fibromyalgia	27	ND	2011	[78]
Childhood acute lymphoblastic leukemia (ALL)	25	ND	2012	[79]
Glaucoma	210	Elevated ADMA in advanced glaucoma	2012	[80]
Pheochromocytoma	18	ND	2013	[81]
Brucellosis	39	ND	2014	[82]
Deep vein thrombosis (DVT)	34	ND	2015	[83]
Short stature	66	ND	2015	[84]
COPD	58	ND	2015	[85]
Nocturia	262	ND	2015	[86]
Neonatal sepsis	31	ADMA correlates with disease severity	2015	[87]
Transient tachypnea of the newborn (TTN)	36	ND	2016	[88]
Arginase 1 deficiency	19	ND	2016	[89]
Idiopathic Parkinson’s disease (PD)	82	ND	2016	[90]

Studies tabulated according to year of first report. ND, not determined.

**Table 2:** Clinical conditions exhibiting elevated SDMA levels.

Patient Population	*N*	Correlation with Clinical Outcome	Year of First Report	Ref.
CKD/ESRD	10	SDMA correlates with renal function	1996	[44,95]
Childhood hypertension	38	SDMA correlates with GFR	1997	[48]
Hyperthyroidism	19	ND	2002	[56]
Critical illness in intensive care unit	52	SDMA correlates with creatinine level	2003	[57]
Hepatorenal syndrome	11	ND	2006	[63]
Coronary artery disease	145	SDMA negatively correlates with GFR	2006	[64]
Type 2 diabetes mellitus (DM)	103	Elevated SDMA in type 2 DM with albuminuria	2007	[96]
Alcoholic hepatitis	52	ND	2007	[97]
Heart failure	132	ND	2008	[98]
Preeclampsia	47	ND	2009	[99]
Stroke	394	SDMA predicts all-cause mortality	2010	[100]
Polycystic ovary syndrome (PCOS)	16	ND	2011	[101]
Glaucoma	210	Elevated SDMA in advanced glaucoma	2012	[80]
Hyperuricemia	58	SDMA correlates with uric acid level	2013	[102]
Malaria	123	ND	2014	[103]

Studies tabulated according to year of first report. ND, not determined.

**Table 3:** Animal models showing intervention with ADMA-lowering effects.

Animal Models	Intervention	Protective Effects	Year of First Report	Ref.
LDL injection-induced endothelial dysfunction in rat	Probucol	Preserve endothelial function	2002	[129]
LDL injection-induced endothelial dysfunction in rat	17β-estradiol	Preserve endothelial function	2004	[130]
Spontaneously hypertensive rat (SHR)	Pioglitazone	Increase renal DDAH-2 expression; Prevent hypertension	2005	[131]
Zucker diabetic fatty rat	Farnesoid X receptor agonist	Increase hepatic DDAH-1 expression; Prevent atherosclerosis	2006	[132]
LDL injection-induced endothelial dysfunction in rat	Taurine	Preserve endothelial function	2007	[133]
Stress-induced preeclampsia in pregnant rat	l-arginine	Prevent hypertension and proteinuria	2008	[134]
SHR	Rosuvastatin	Attenuate hypertension	2008	[135]
STZ-induced diabetic rat	Telmisartan	Reduce renal PRMT-1 expression; Increase renal DDAH-1 expression	2008	[136]
Ethanol-induced gastric mucosal injury in rat	Resveratrol analog BTM-0512	Prevent gastric mucosa injury; Increase DDAH activity	2010	[137]
Bile duct-ligated cirrhotic rat	Melatonin	Prevent liver damage; Increase DDAH activity	2010	[117]
SHR	Melatonin	Prevent hypertension; Increase DDAH activity	2010	[138]
SHR	Aliskiren	Prevent hypertension	2011	[139]
SHR	Nebivolol	Prevent hypertension	2011	[140]
Monocrotaline-induced pulmonary hypertension in rat	Rosuvastatin	Prevent pulmonary hypertension	2011	[141]
Bile duct-ligated cirrhotic rat	Ornithine phenylacetate	Prevent liver damage	2012	[142]
High-fat diet in rat	Atorvastatin	Improve endothelial function; Increase DDAH activity	2012	[143]
5/6 nephrectomized rats	Shichimotsukokato	Prevent hypertension; Increase DDAH-2 level	2012	[144]
Bile duct-ligated cirrhotic rat	Vitamin E	Improve endothelial function; Increase hepatic DDAH-2 level	2012	[145]
Coronary artery-ligated rat	Salvianolic acid A	Improve cardiac damage; Increase DDAH activity	2013	[146]
STZ-induced diabetic pregnant rat	l-citrulline	Prevent offspring hypertension; Increase renal DDAH-2 level	2013	[112]
STZ-induced diabetic rat	Glucagon-like peptide-1 receptor agonist	Protect diabetic nephropathy; Reduce PRMT-1 expression	2013	[147]
SHR	*N*-acetylcysteine	Prevent gastric mucosa injury; Increase DDAH activity	2013	[148]
Prenatal dexamethasone exposure in rat	l-citrulline	Prevent offspring hypertension	2014	[149]
SHR	l-citrulline	Prevent hypertension	2014	[150]
SHR	Sodium nitrate	Prevent hypertension	2014	[150]
High-fat and high-cholesterol diet in rat	Atorvastatin plus rosiglitazone	Protect endothelial function	2014	[151]
Isoproterenol-induced heart failure in rat	Oxymatrine	Ameliorate ventricular function and hypertrophy; Increase DDAH-2 expression	2014	[152]
Angiotensin II-induced hypertension in rat	Serelaxin	Attenuate hypertension and proteinuria	2014	[153]
Lipopolysaccharide/D-galactosamine-induced liver injury	Metformin	Protect liver injury/Increase DDAH activity	2014	[154]
SHR	Metformin	Prevent hypertension	2014	[110]
Maternal caloric restriction.rat	Melatonin	Prevent offspring hypertension	2014	[155]
Constriction of artery-induced subarachnoid hemorrhage in rat	18β-glycyrrhetinic acid	Improve neurological outcome	2015	[156]
Myocardial ischemia/reperfusion injury in rat	Apocynin	Protect myocardial injury	2015	[157]
Maternal caloric restriction.rat	Aliskiren	Prevent offspring hypertension	2015	[158]
High-fat and high-cholesterol diet in rat	Atorvastatin	Protective endothelial function	2015	[159]
10% furctose administration rat	Fenofibrate	Reduce triglyceride level	2015	[160]
Cyclosporine-induced nephrotoxicity	Nebivolol	Ameliorate endothelial function	2016	[161]
l-NAME induced hypertension in rat	Novokinin	Prevent hypertension	2016	[162]
Bile duct-ligated cirrhotic rat	Etanercept	Prevent brain damage	2016	[163]
			2016	[164]
STZ-induced cognitive impairment in rat	H_2_S releasing compounds ATB-346 and diallyl trisulfide	Ameliorate behavior performance	2016	[165]
Aged rat	Epigallocatechin-3-gallate	Ameliorate erectile function; reduce PRMT-1 expression; Increase DDAH activity	2016	[166]
Prenatal dexamethasone plus postnatal high-fat diet in rat	*N*-acetylcysteine	Prevent hypertension	2016	[167]
Isoproterenol-induced heart failure in rat	Rosuvastatin	Ameliorate ventricular function and hypertrophy; Reduce PRMT-1 expression; Increase DDAH-2 expression	2016	[168]

Studies tabulated according to year of first report.
